# Safety and Blood Loss Associated With Tourniquets in Total Knee Arthroplasty

**DOI:** 10.7759/cureus.16875

**Published:** 2021-08-04

**Authors:** Orfan M Arafah, Abdullah M Alotaibi, Ahmed M Alsalloum, Hatim M Alotaibi

**Affiliations:** 1 Orthopaedic Surgery, King Khalid University Hospital, Riyadh, SAU; 2 Medicine, King Khalid University Hospital, Riyadh, SAU

**Keywords:** total knee arthroplasty, total knee replacement, tourniquet, total blood loss, thromboembolic events

## Abstract

Objective

To measure total blood loss and safety of tourniquets, tourniquets during cementation, or no tourniquets during total knee arthroplasty (TKA).

Methods

This retrospective cohort study included 75 patients from King Khalid University Hospital in Riyadh, Saudi Arabia. Patients were equally divided into three groups: tourniquets, no-tourniquets, and tourniquets during cementation. Recorded data included: baseline characteristics (age, sex, weight, height, body mass index (BMI), anesthesia type, and comorbidities), blood loss parameters (total blood loss, preoperative and postoperative hematocrit (Hct) and hemoglobin (Hgb), and blood transfusion units), duration of surgery, hospital stay, and thromboembolic events during 3-months postoperatively. Statistical significance was reported by using a P-value < 0.05, and 95% confidence intervals.

Results

The tourniquet group had significantly lower mean total blood loss (544.67 mL ± 418.86; P = 0.001), higher mean postoperative hemoglobin values (115.44 g/dL ± 10.97; P = 0.004) and hematocrit (34.25 % ± 3.35; P = 0.005), and lower median intra-operative loss (100 cc, 10-300; P < 0.001), than the other groups. Tourniquets during cementation required significantly more mean surgery time (131.84 minutes ± 22.12; P = 0.003), and longer median hospital stay (8 days, 5-13) than the other groups.

Conclusion

Tourniquet use during TKA significantly decreased total and intraoperative blood loss, but it did not have any significant effect on the transfusion rate or the incidence of thromboembolic events.

## Introduction

Total knee arthroplasty (TKA) has been routinely performed for decades, and its use continues to increase worldwide [1]. TKA is commonly indicated for severe primary osteoarthritis in case of nonsurgical treatment failure [2,3].

During TKA, tourniquets are often used to enhance visualization and shorten surgery times by creating bloodless fields for orthopedic surgeons [4,5]. Nonetheless, the efficacy and safety of tourniquets in TKA remains a controversial topic.

Patients who undergo TKA procedures can suffer from significant blood loss and be at risk for subsequent blood transfusions [6,7]. Tourniquet usage is suggested to decrease blood loss [6]. However, the results on blood loss in TKA with and without tourniquet are contradictory, and the question remains unsolved [6,7].

Blood loss is often described as intraoperative, postoperative, and hidden blood loss [6]. Total blood loss is the sum of the previously mentioned blood loss types, which can be measured in different ways. Gross’s formula is a common method, which uses the difference in hemoglobin as a main variable for calculating the total blood loss [8].

Due to the lack of clear evidence on the role of tourniquets in total blood loss during TKA, we conducted this retrospective cohort study primarily to measure the total blood loss with tourniquets, tourniquets during cementation, and without tourniquets during TKA.

## Materials and methods

Study participants

This retrospective cohort study included 75 patients from King Khalid University Hospital in Riyadh, Saudi Arabia. Patients of both sexes undergoing TKA with tourniquet during the entire surgery, during cementation only or without tourniquet were included. Patients were excluded if they had undergone bilateral surgery, previous TKA revision, had a hemorrhagic disorder, or ongoing use of anticoagulant drugs at the time of surgery. At a level of significance of 0.05, a power of 0.95, and a large effect size = 0.47, the estimated sample size was 75. The participants were selected using a computerized simple random technique, patients undergoing TKA during the period of January 2019 to December 2020 were divided into three groups, tourniquets group (tourniquets during the whole surgery), no-tourniquets group (no tourniquets during surgery), and tourniquets during cementation (tourniquets only during cementation), a computer-generated number was given to each participant, 25 participants were selected randomly for each group

Study procedures

In this study, patients recorded data included: baseline characteristics (age, sex, weight, height, body mass index (BMI), anesthesia type, and comorbidities), blood loss parameters (total blood loss, preoperative and postoperative hematocrit (Hct) and hemoglobin (Hgb), and blood transfusion units), duration of surgery, hospital stay, and thromboembolic events during three-months postoperatively.

Ethical considerations

The Institutional Review Board at the College of Medicine, King Saud University approved this study. Informed written consent was provided by all participants.

Statistical analyses

Data were analyzed using the IBM SPSS Statistics for Windows, version 26.0 (IBM Corp., Armonk, NY, USA). Frequencies and percentages are used to describe categorical variables. We used Shapiro -Wilk test for normality testing. Mean and standard deviation were used to describe continuous variables with normal distribution, comparison was done using one-way ANOVA. Median and interquartile ranges were used to describe continuous variables with abnormal distribution, comparison was done using Kruskal-Wallis test. We used as a post hoc test Tukey’s HSD test for one-way ANOVA, and Mann-Whitney U test for Kruskal-Wallis test. Statistical significance was reported by using a P-value < 0.05, and 95% confidence intervals.

## Results

A total of 75 patients who had undergone TKA were recruited. The mean age was similar between groups. Although most patients were female, there were slightly more female patients in the no-tourniquet group than in the other groups. There was no major disparity among the groups with regards to baseline comorbidities. All other characteristics are listed in Table 1.

**Table 1:** Characteristics of patients undergoing total knee arthroplasty (N = 75). *Statistically significant P < 0.05. BMI: body mass index.

		Tourniquet group (n = 25)	No-tourniquet group (n = 25)	Tourniquet during cementation group (n = 25)	P-value
Age (years)		63.36 ± 7.4	64.36 ± 7.66	64.92 ± 9.63	0.792
Sex	Males	7 (28%)	3 (12%)	9 (36%)	0.139
	Females	18 (72%)	22 (88%)	16 (64%)	
BMI		33.98 ± 5.5	34.05 ± 6.25	32.99 ± 6.21	0.784
Anesthesia type	General	9 (36%)	13 (52%)	10 (41.7%)	0.425
	Spinal	15 (60%)	9 (36%)	14 (54.2%)	
	Epidural	1 (4%)	3 (12%)	1 (4.2%)	
Co-morbidities	Diabetes mellitus	18 (72%)	12 (48%)	16 (64%)	0.207
	Hypertension	11 (44%)	13 (52%)	13 (52%)	0.808
	Dyslipidemia	7 (28%)	8 (32%)	2 (8%)	0.095
	Others	5 (16%)	5 (20%)	6 (20%)	0.924

Normality assumptions were tested using the Shapiro-Wilk test for independent variables (total blood loss, preoperative and postoperative Hct and Hgb values, transfusion units, duration of surgery, and hospital stay) among groups (tourniquets, no-tourniquet, tourniquets during cementation). Only two independent variables (intraoperative blood loss and hospital stay) did not meet the assumption of a normal distribution. Hence, the Kruskal-Wallis test was used to test the statistical significance of these variables among groups. The remaining variables were tested using a one-way ANOVA.

The results of the one-way ANOVA and Kruskal-Wallis tests are presented in Table 2. There was a statistically significant difference in mean total blood loss among the three groups (P = 0.001). The tourniquet group had significantly lower mean total blood loss (544.67 mL ± 418.86) than no-tourniquet (833.36 mL ± 352.05) and tourniquet during cementation groups (920.51 mL ± 317.97).

**Table 2:** Blood loss parameters. *Statistically significant at P < 0.05. Hct: hematocrit; Hgb: hemoglobin.

		Tourniquet group (n = 25)	No-tourniquet group (n = 25)	Tourniquet during cementation group (n = 25)	F-value	P-value	Post hoc test			
		Mean ± SD	Mean ± SD	Mean ± SD			Tourniquet group			
								Mean difference	P-value	
Total blood loss (ml)		544.67 ± 418.86	833.36 ± 352.05	920.51 ± 317.97	7.250	0.001	No-tourniquet	-288.69	0.018	
							During cementation	-375.84	0.001	
Intra-operative blood loss (cc)		Median = 100, range (10-300)	Median = 200, range (50-400)	Median = 200, range (100-500)	15.383 (chi-square)	<0.001	No-tourniquet	-3.47 (Z score)	0.001	
							During cementation	-3.29 (Z score)	0.001	
Pre-operative values:	Hct (%)	39.69 ± 2.7	38.57 ± 4.37	38.37 ± 4.12	0.047	0.954				
	Hgb (g/dl)	129.4 ± 9.89	127.61 ± 14.76	127.16 ± 13,58	0.210	0.811				
Postoperative values:	Hct (%)	34.25 ± 3.35	31.56 ± 3.93	31.07 ± 3.35	5.795	0.005	No-tourniquet	2.69	0.025	
							During cementation	3.17	0.006	
							No-tourniquet	8.66	0.027	
	Hgb (g/dl)	115.44 ± 10.97	106.77 ± 12.28	104.92 ± 11.49	5.861	0.004				
							During cementation	10.52	0.006	
Transfusion (unit)		-	-	-	2 (chi-square)	0.368				

Intraoperative blood loss was statistically significant among groups (P < 0.001). The tourniquet group had a significantly lower median intraoperative blood loss (100 cc, 10-300) than no-tourniquet (200 cc, 50-400) and tourniquet during cementation groups (200 cc, 100-500).

There was a statistically significant difference between groups in the mean postoperative of Hgb and Hct values (P = 0.004 and P = 0.005, respectively). The tourniquet group had statistically significant higher mean postoperative Hgb (115.44 g/dL ± 10.97) and Hct (34.25 % ± 3.35) than no-tourniquet Hgb (127.61 g/dL ± 14.76) and Hct (38.57 % ± 4.37) and tourniquet during cementation group Hgb (104.92 g/dL ± 11.49) and Hct (31.07 % ± 3.35). However, the preoperative Hct and Hgb values were not significantly different between groups. Further, the transfusion rate was not significantly different among groups (Table 2).

The mean surgery time was significantly different among groups (P = 0.003). The tourniquet during cementation required a significantly longer mean surgery time (131.84 minutes ± 22.12) than tourniquet (111.44 minutes ± 18.89) and no-tourniquet groups (111.76 minutes ± 27.99).

Further, there was a significant variance in the median hospital stay (P < 0.001), with tourniquet during cementation group having a significantly higher median hospital stay (8 days, 5-13) than tourniquet (5 days, 3-15) and no-tourniquet groups (6 days, 3-9). However, the incidence of thromboembolic events was not significantly different among groups (Table 3).

**Table 3:** Surgery time, hospital stay and thromboembolic events. *Statistically significant at P < 0.05.  DVT: deep vein thrombosis; PE: pulmonary embolism.

	Tourniquet group (n = 25)	No-tourniquet group (n = 25)	Tourniquet during cementation group (n = 25)	F-value	P-value	Post hoc test		
	Mean ± SD	Mean ± SD	Mean ± SD			During cementation group		
							Mean difference	P-value
Surgery time (minutes)	111.44 ± 18.89	111.76 ± 27.99	131.84 ± 22.12	6.287	0.003	No-tourniquet	20.08	0.009
						Tourniquet	20.4	0.008
Hospital stay (days)	Median = 5, range (3-15)	Median = 6, range (3-9)	Median = 8, range (5-13)	19.120 (chi-square)	<0.001	No-tourniquet	-3.16(Z score)	0.002
						Tourniquet	-4.09 (Z score)	<0.001
DVT	-	-	-	2 (chi-square)	0.368			
PE	-	-	-	0 (chi-square)	1.00			

## Discussion

Our results emphasize the overall recommendations for the use of tourniquets during TKA. Further, our results helped clarify the question of total blood loss with tourniquet use, as they revealed that patients who underwent TKA with tourniquets had less total blood loss than patients without tourniquets or with tourniquets during cementation only. Contrary to our results, some studies have suggested that tourniquet use does not have any benefits regarding total blood loss [4,9-10]. However, our finding is in line with many studies that concluded a beneficial effect of tourniquets over other approaches with regards to total blood loss [5,11-14]. This discrepancy could be due to the diversity in the assessment methods for total blood loss measurements in the literature. Therefore, there is a need to re-evaluate these questions in high-quality meta-analysis.

Our results indicate that intraoperative blood loss was significantly lower with tourniquet use during the entire surgery compared to the other techniques. In agreement with recent reviews which showed that the use of tourniquets significantly reduces intraoperative loss [4,6,11]. We, therefore, consider that the effect of tourniquets on intraoperative blood loss is no longer a research question.

Although there were no differences in baseline preoperative Hgb and Hct values, the postoperative values were significantly higher in patients with tourniquets during the entire surgery. These findings strengthen the evidence toward the use of tourniquets during TKA surgery.

Although total blood loss and intraoperative blood loss were significantly lower with tourniquets, our study did not find any significant differences in transfusion rates between groups. This is consistent with the results of a recent meta-analysis [4,10-11].

Regarding the risk associated with tourniquet use during TKA, recent studies did not find any association between tourniquet use and the risk of thromboembolic events [15-17]. Our findings are in agreement with these studies. However, the incidence of thromboembolic events after TKA varies from 1­% to 84% [18]. This variation is likely due to the heterogeneity in study groups and the discrepancy in the methods used to evaluate the incidence in the literature. Therefore, high-quality prospective studies with larger sample sizes are needed to measure the incidence of thromboembolic events and evaluate the factors associated with these fatal events.

Surgery time and hospital stay days were significantly higher in association with tourniquets during cementation. This is a contradiction with the results of a recent meta-analysis [9]. this could be attributed to the heterogeneity of surgical techniques, perioperative care and anaesthetic protocol [1,3,9].

Limitations

This is a retrospective study and has the potential to include inaccurate data. Second, the procedures were performed by multiple surgeons, which may affect the results depending on the surgeon’s skills. Third, the sample size was small, which decreases the statistical power and has the potential for bias.

## Conclusions

Tourniquet usage during TKA significantly decreases total and intraoperative blood loss, but it does not have a significant effect on the transfusion rate or incidence of thromboembolic events. The use of tourniquet only during cementation is associated with longer surgery time and longer hospital stay. High-quality research is needed to confirm the safety of tourniquets during TKA.
